# Magnetic Resonance Imaging of Paediatric Orbital Tumors With Pathological Correlation

**DOI:** 10.7759/cureus.86255

**Published:** 2025-06-17

**Authors:** Deepak Kumar, Umakant Prasad, Rashmi R Bharti, Sulekha Kumari, Amit Kumar, Gyan Bhaskar, Rizwan Ahmar

**Affiliations:** 1 Radiodiagnosis, Indira Gandhi Institute of Medical Sciences, Patna, Patna, IND; 2 Pathology, Indira Gandhi Institute of Medical Sciences, Patna, Patna, IND; 3 Radiology, Indira Gandhi Institute of Medical Sciences, Patna, Patna, IND; 4 Ophthalmology, Indira Gandhi Institute of Medical Sciences, Patna, Patna, IND; 5 Pediatric Medicine, Indira Gandhi Institute of Medical Sciences, Patna, Patna, IND

**Keywords:** ocular dermoid, orbital mri, orbital tumour, pediatric orbit, retinoblastoma

## Abstract

Introduction: Paediatric orbital tumours include a broad spectrum of benign and malignant lesions that range from developmental anomalies to primary and metastatic orbital malignancies. Sometimes, clinical signs and symptoms are not enough to differentiate between orbital lesions; hence, MRI plays a crucial role in cases where clinical and historical findings are inconclusive.

Aims and objectives: The aim of the study is to evaluate the different orbital tumours in paediatric patients by magnetic resonance imaging and their pathological correlation.

Materials and methods: We conducted a prospective observation study of 64 paediatric patients (38 males and 26 females), with an age range of one month to 15 years and a mean of 4.6 years, who underwent orbital MRI for suspected tumors at Indira Gandhi Institute of Medical Science, Patna, a tertiary care center, over a period of one year.

Results: Of 64 patients (mean age 4.6 years), 38 (59.3%) were boys and 26 (40.7%) were girls. The most common presentation in our study is leukocoria (white pupillary reflex), and out of 64 cases, 37 (57.8%) were found malignant and 27 (42.2%) were benign lesions. The most common diagnosis was retinoblastoma (32 cases, 50%). Common benign tumours include dermoid cyst, capillary hemangioma, epidermal inclusion cyst and lipodermoid cyst.

Conclusion: As symptoms are not well appreciated in paediatric orbital tumours, for early diagnosis and staging, excellent imaging (MRI ) and histopathological examination are necessary in order for timely detection and to prevent advanced stage at presentation.

## Introduction

Pediatric orbital tumours include a broad spectrum of benign and malignant lesions, ranging from developmental anomalies to primary and metastatic orbital malignancies. Sometimes, clinical signs and symptoms are not enough to differentiate between orbital lesions; hence, magnetic resonance imaging (MRI) plays a very important role in their diagnosis and management, where clinical and history are inconclusive [1].

Ultrasound is the primary investigation but has a limited role in the evaluation of retrobulbar disease extent and bony destruction [2]. CT is a good modality for bony destruction, but due to the risk of carcinogenesis effects of radiation in the paediatric population, it is avoided. MRI is the preferred modality, providing excellent soft tissue contrast and multiplanar capability without ionising radiation [3]. MRI can characterise lesion signal properties, enhancement patterns, vascular flow and full anatomic extent within the orbit, which helps differentiate tumour types and guide management.

## Materials and methods

We performed a prospective observational study on 64 patients with suspected orbital tumours who underwent orbital MRI at Indira Gandhi Institute of Medical Sciences, India, from June 2023 to May 2024. Institutional Ethics Committee approval (ref. no. 1010/IEC/IGIMS/2023) and parental consent were obtained.

The patients mostly presented with leukocoria and proptosis. MRI was done for all 64 patients, followed by biopsy for histopathological study. The study population was all paediatric patients below 15 years of age with suspected orbital tumours. An MRI orbit with or without contrast was done in all patients. Patients who had a suspected ferromagnetic implant or metallic foreign body in the body were excluded from the study. Patients who were claustrophobic and whose MRI images were sub-optimal due to motion/ other artefacts were also excluded.

Equipment

Forty-two patients were imaged on a 1.5 Tesla superconducting MRI machine (GE made, Model Optima MR360), and 22 patients were imaged on a 1.5 Tesla superconducting MRI machine (GE made, Model Optima MR450W GEM and SIGNA Artist system) by using an eight-channel head coil. For optimal imaging, patients have to remain motionless, and infants-children were sedated by either oral or IV drugs under the presence of a paediatrician or anaesthetist. Consent of the parent or guardian was taken before the procedure. Generally, oral syrup Pedicloryl (triclofos sodium) with a dosage of 25 mg/kg/dose was given. Short-acting general anaesthesia was given under supervision to those who were not getting sedated by oral drugs. 

Axial, coronal and sagittal T2-weighted sequences and coronal and axial T1-weighted images were acquired. Additional sequences included axial T2-weighted fat-suppressed, axial diffusion-weighted imaging, sagittal oblique T1 and axial/coronal fat suppression T1 after gadolinium administration. 

All patients also underwent pathological diagnosis either by fine-needle aspiration (FNAC) or biopsy (tru-cut, incisional, and excisional). Imaging and histopathology results were recorded for each case.

Statistical analysis was performed using IBM SPSS Statistics for Windows, version 22.0 (released 2013, IBM Corp., Armonk, NY). Descriptive statistics (mean, percentages, frequencies) summarised the data. Categorical comparisons (e.g., sex distribution by tumour type) were evaluated using the chi-square test. A p-value <0.05 was considered statistically significant. 

## Results

The pool of this study is 64 patients, with 38 males (59.3%) and 26 females (40.7%). The age group ranged from one month to 15 years, the mean age was 4.6 years, and the most affected age group was one to five years.

**Figure 1:**
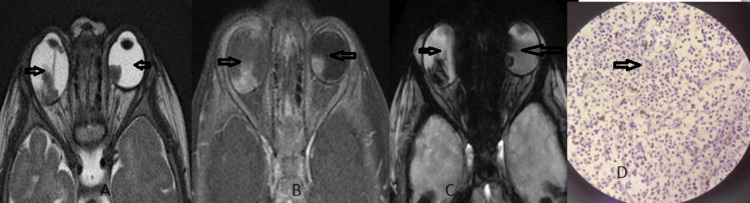
Retinoblastoma Axial T2W (A) image show triangular hypointensity in the posterior aspect of the bilateral globe (open arrow) with a persistent hyaloid membrane in the right eye and lesion showing enhancement on the axial T1W FS post contrast study (B), bilateral globe lesion bloom axial GRE sequence due to calcification (C), and image (D) show the histopathological finding of retinoblastoma.

**Figure 2:**
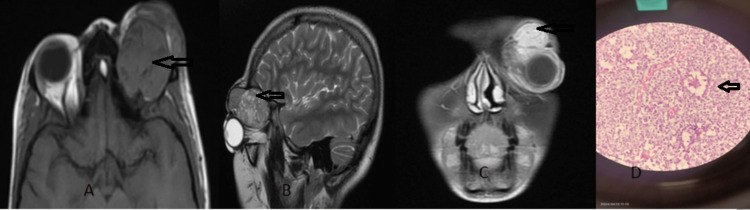
Capillary hemangioma An extraconal lesion in the left orbit shows an isointense signal intensity on  axial T1W (A), heterogenous hyperintense signal intensity on sagittal T2W (B) and homogenous enhancement in the coronal T1 fat-suppressed contrast-enhanced  image (C), and image (D) show the histological finding of capillary hemangioma.

**Figure 3:**
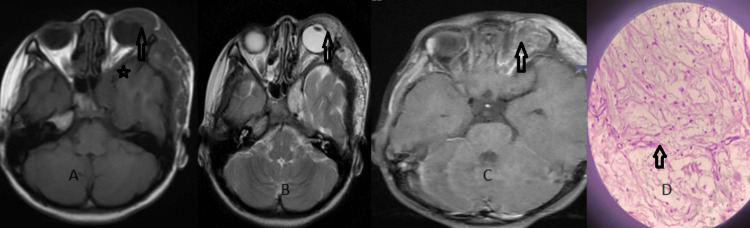
Plexiform neurofibroma Axial T1W (A) shows a heterogeneously hypointense lesion involving the preseptal tissue and superolateral extraconal component of the left orbit, which extends along the left temporal calvarium. The lesion appears heterogeneously hyperintense on T2W (B) and shows heterogenous enhancement on the T1 fat-saturated contrast-enhanced image (C) . There is sphenoid wing dysplasia also noted on the left side (star mark). Image (D) shows the histological finding of neurofibroma.

The primary complaint was leukocoria, followed by protrusion of the eye. Out of 64 cases, 37 (57.8%) were malignant and 27 (42.2%) were benign (Table 1). The three most common tumours included retinoblastoma (32 cases, 50%), orbital dermoid cyst (nine cases, 14%), and capillary hemangioma (seven cases, 11%). Among the 37 malignant tumours, retinoblastoma is the most common (32 cases, 84.2%). MRI and histopathological findings are shown in Figure 1. Among the benign tumours, the three most common include dermoid cyst (nine cases, 33%), capillary hemangioma (seven cases, 26%) (Figure 2), and epidermal inclusion cysts (four cases, 14.8%), and orbital dermoid cyst is the most common benign tumour (Table 2). Plexiform neurofibroma associated with NF1 was seen in four patients (Figure 3).

**Table 1:** Distribution of the nature of tumors (malignant vs. benign) by sex. Data are presented as N(%). The chi-square test was used for comparison (X2 = 0.62, p = 0.430, significance threshold p < 0.05).

Nature of disease	Males (N,%)	Female (N, %)	Total	Chi-square test statistic	p-value
Malignant	24 (63.2%)	13 (50%)	37 (57.8%)	X^2 ^= 0.62	p = 0.430
Benign	14 (36.8%)	13 (50%)	27 (42.2%)		
Total	38 (59.3%)	26 (40.7%)	64 (100%)		

**Table 2:** Frequency of various orbital tumors in MRI by sex. Data are presented as N (%). The chi-square test was used for comparison (X2 = 7.62, p = 0.472, significance threshold p < 0.05).

Diagnosis	Males (N, %)	Females (N, %)	Total	Chi-square test statistic	p-value	
Retinoblastoma	19 (50%)	13 (50%)	32 (50%)	X^2 ^= 7.62	P=0.472	
Capillary hemangioma	3 (7.9%)	4 (15.4%)	7 (11%)			
Plexiform neurofibroma	1 (2.6%)	1 (3.8%)	2 (3.15%)			
Rhabdomyosarcoma	2 (5.3%)	0 (0%)	2 (3.15%)			
Dermoid cyst	6 (15.7%)	3 (11.6%)	9 (14%)			
Lipodermoid	2 (5.3%)	1 (3.8%)	3 (4.65%)			
Orbital infiltration in leukemia and Mets:	3 (7.9%)	0 (0%)	3 (4.65%)			
Epidermal inclusion cyst	2 (5.3%)	2 (7.7%)	4 (6.25%)			
Optic nerve glioma	0 (0.0%)	2 (7.7%)	2 (3.15%)			
Total	38 (59.3%)	26 (40.7%)	64 (100%)			

Sixty out of 64 patients showed the same results on the MRI and pathological examination, which reflects that MRI is 93.75% sensitive in the diagnosis of orbital tumours. The discordance between MRI and histopathological diagnosis was noted in four (6.5 %) patients; however, there is no discordance in the nature of disease, i.e., all discordant diseases are benign in the MRI and histopathologically (Table 3). 

**Table 3:** Discordance between the MRI finding and histopathology.

MRI finding	Histopathology	Total	Nature of disease (MRI and HPE)
Dermoid	Lipo-dermoid	1	Benign
Dermoid	Epidermal inclusion cyst	1	Benign
Optic nerve glioma	Ganglioglioma	2	Benign

Two MRI-diagnosed dermoid cysts became lipo-dermoid and epidermal inclusion cysts, respectively, on the pathology. Two lesions interpreted as optic nerve gliomas on the MRI were proven to be gangliogliomas histologically.

## Discussion

Paediatric orbital tumour is about 2% of all tumours. Our study shows that 37 cases (57.8%) are malignant and 27 cases (42.2%) are benign, which are concordance with Mohan et al.'s report (a study of 68 orbital tumors) with incidences of 28% and 72% of benign and malignant tumors, respectively, and also agree with many prior studies. 

Our finding that retinoblastoma came as the most common overall tumour (32 cases, 50%), i.e., retinoblastoma accounted for half of all orbital lesions, which agrees with many prior studies [4]. The mean age of presentation for retinoblastoma in our study was 40 months, similar to reports from a Kenyan study, in which the reported mean age of retinoblastoma is 39.9 months [5]. An Ethiopian study reported the mean age for the right eye of 34.4 and left eye of 40.2 months [4,5].

Twenty-nine cases (90.5%) of retinoblastoma are unilateral, and three cases (9.5%) show bilateral retinoblastoma. Trilateral retinoblastoma is nil in the study. MRI was used to confirm the diagnosis and for tumour staging. MRI is the most sensitive technique for evaluating retinoblastoma and staging, especially regarding tumour infiltration of the optic nerve, extraocular extension, and intracranial disease [6,7,8,9]. In MRI, heavily T2W sequences (FIESTA-high spatial resolution imaging) and small intra-ocular tumours are best detected. To see the calcification in retinoblastoma, computed tomography is not essential, so we should try to avoid the use of computed tomography due to ionising radiation.

**Figure 4:**
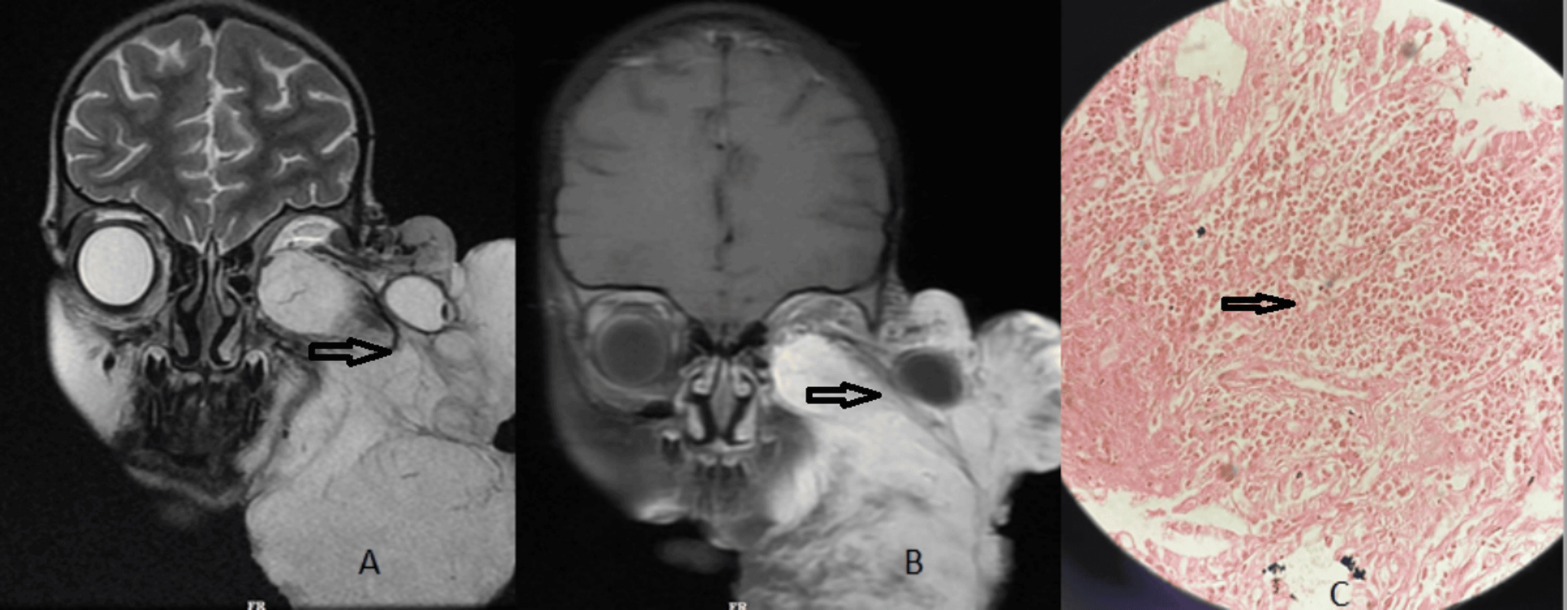
Rhabdomyosarcoma Coronal (A) T2W MRI image with contrast image (B) shows a large enhancing soft tissue mass involving the left orbit with external protrusion of the left orbit (open arrow) and image (C) show the histological finding of rhabdomyosarcoma.

Rhabdomyosarcoma is the most common mesenchymal tumour of the head and neck in childhood, with 10% of all cases occurring in the orbit and usually presenting with rapidly progressive proptosis [10]. It is an extraconal tumour that arises from extraocular muscles or the eyelid (Figure 4). It typically presents with rapid unilateral proptosis in young children. Embryonal rhabdomyosarcoma is the most common, accounting for 71% and 67% of cases of orbital rhabdomyosarcoma [11,12]. It has an excellent prognosis.

Capillary haemangioma is the most common benign orbital tumour in children [13]. It can be detected a few weeks after birth [14]. The most common location is the superomedial extraconal space, but it can be intraconal or periorbital. It shows a predictive evolution pattern, as proliferative rapid enlargement for several months, followed by a brief plateau phase and finally involuted by one to two years of age. Small superficial lesions can be assessed by ultrasound. MRI is usually needed for atypical or extensive hemangioma (Figure 2).

A distinct finding in some patients was plexiform neurofibroma (two cases, 3.15%) associated with neurofibromatosis-1. It presents as isolated cases or is associated with Von Recklinghausen disease. Plexiform neurofibroma is pathognomonic of NF1. It almost occurs exclusively in children with NF-1. MRI helps in defining the extent of the lesion. It can be seen within the orbit, periorbital region, scalp, temporal fossa, and skull base. MRI demonstrates low signal intensity on T1W and moderate-high signal intensity on T2W (Figure 3) and heterogeneity of signal strength within the lesion, reflecting the mixed histopathology and vascularity of the tumours [15].

Orbital metastasis is most common in the case of neuroblastoma, followed by leukaemia. Orbital infiltration occurs in the form of myeloid sarcoma in patients with AML. These are mostly extraconal. If the optic nerve gets involved, it is a medical emergency.

The limitation of our study is the small sample size and possible referral bias since it is a single-centre (hospital-based) study. The strength of the study is increased by increasing the number of patients. Nonetheless, by combining imaging and pathology, we provide a comprehensive overview of paediatric orbital tumours in our setting.

## Conclusions

In this study of 64 cases of paediatric orbital tumours, 37 cases (57.8%) were malignant and 27 cases (42.2%) were benign. Retinoblastoma is the most common orbital neoplastic lesion. MRI proved highly effective, with concordant imaging-pathology diagnosis in 94% of cases. Our findings underscore that early MRI evaluation, along with tissue diagnosis, is critical for accurate classification and staging of childhood orbital tumours. Clinicians should maintain a high index of suspicion for retinoblastoma and other aggressive lesions in children presenting with leukocoria or proptosis. A multidisciplinary approach integrating detailed MRI characteristics and histopathology allows timely diagnosis and guides appropriate therapy to improve outcomes in pediatric orbital neoplasms.
